# Supplementary material for: Protocol for a scoping review exploring the use of patient-reported outcomes in adult social care
Source: BMJ Open. 2021 Apr 13;11(4):e045206. doi: 10.1136/bmjopen-2020-045206 (PMC8051391; doi:10.1136/bmjopen-2020-045206)
Supplement: Supplementary data [file bmjopen-2020-045206supp001.pdf]

Supplemental File 1 – Search strategy

Note: Lines 48-72 are based on the modified Oxford PROM filter and the search strategy developed by Aiyegbusi et al.[1]

	**Setting**
1	respite care/
2	respite care.ti,ab.
3	hospice/ or home care/ or palliative care/ or terminally ill patients/
4	exp client transfer/ or exp facility discharge/ or exp hospital discharge/ or exp patient selection/ or exp professional referral/ or exp psychiatric hospital discharge/ or exp treatment refusal/ or exp treatment termination/
5	patient transfer.ti,ab.
6	hospice.ti,ab.
7	residential home.ti,ab.
8	care home*.ti,ab.
9	aged care home*.ti,ab.
10	nursing homes/
11	retirement home*.ti,ab.
12	adult day care centre*.ti,ab.
13	homes for the aged.ti,ab.
14	self-care skills/ or aging in place/ or independent living programs/ or rehabilitation/
15	independent living.ti,ab.
16	personalised budget*.ti,ab.
17	reablement.ti,ab.
18	home nursing.ti,ab.
	**Population**
19	aged.ti,ab.
20	geriatric patients/
21	old* person.ti,ab.
22	(elderly or geriatric).ti,ab.
23	exp Disabilities/ or exp Intellectual Development Disorder/ or exp Developmental Disabilities/
24	disabled person*.ti,ab.
25	disabled people.ti,ab.
26	(disabled adj3 person*).ti,ab.
27	vulnerable population*.ti,ab.
28	vulnerable person*.ti,ab.
29	intellectual development disorder/
30	exp Autism Spectrum Disorders/
31	autism spectrum disorder.ti,ab.
32	intellectual disabilit*.ti,ab.
33	learning disabilit*.ti,ab.
34	exp Chronic Illness/ or exp Long Term Care/

35	long term condition*.ti,ab.
36	chronic illness.ti,ab.
37	exp substance-related disorders/
38	mental* ill*.ti,ab.
39	drug user.ti,ab.
40	homeless.ti,ab.
41	clients/
42	service user*.ti,ab.
43	adult.ti,ab.
	**Type of Care**
44	exp Social Services/
45	social care.ti,ab.
46	integrated services/
47	integrated care.ti,ab.
	**PROs**
48	(PRO integration or Clinical PRO application* or telePRO or automated PRO algorithm* or screening purpose* or PRO questionnaire* or Patient-reported outcome questionnaire* or PROM or Patient-reported outcome measure* or Patient reported outcome measure* or Patient-reported symptom* or Patient-centred care or Patient self-report* or Self-report health or Self-rated health or Self-reported measure* of health or Health outcome* or Health communication* or Hospital performance evaluation* or Automated telephone survey system* or paper-based survey* or web-based survey* or web-based PRO platform* or web-based system* or PRO collection* or PRO measure* or PRO intervention* or PRO assessment intervention* or PRO data or PRO assessment* or Routine PRO assessment* or Routine PRO collection or Symptom assessment* or Symptom monitoring or Symptom data or Functional status or Electronic PRO assessment* or Electronic PRO system* or ePRO or ePRO* or ePRO system* or PRO system* or Generic PRO system* or PRO-based clinical alert system*).ti,ab.
49	exp Patient Reported Outcome Measures/
50	((((patient adj reported adj outcome adj measure*) or patient) adj reported adj outcome*) or capability or capabilities).ti,ab.
51	((disability or function or functional or functions or subjective or utility or utilities or wellbeing or well being) adj2 (index or indices or instrument or instruments or measure or measures or questionnaire* or profile or profiles or scale or scales or score or scores or status or survey or surveys)).ti,ab.
52	((patient or self or carer or proxy) adj (appraisal* or appraised or report or reported or reporting or rated or rating or based or assessed or assessment*)).ti,ab.
53	self-report/
54	health status/
55	(health index* or health indices or health status or health profile*).ti,ab.
56	exp "Quality of Life"/
57	quality of life.ti,ab.
58	(HR-PRO or HRPRO or HRQL or HRQoL or QL or QoL).ti,ab.

59	(Patient Centred Care or person centred care or person centred coordinated care or p3c or person centred outcome*).ti,ab. or person reported outcome*.mp. or individualised patient reported outcome measur*.ti,ab. or iprom.ti,ab.
60	patient centered care.ti,ab.
61	person centered care.ti,ab.
62	(Patient Centered Care or person centered care or person centered coordinated care or p3c or person centred outcome*).ti,ab. or person reported outcome*.mp. or individualised patient reported outcome measur*.ti,ab. or iprom.ti,ab.
	**Implementation**
63	((implementation or implementation science).ti,ab. or implementation.mp.) adj3 science.ti,ab.
64	"barriers and facilitators".ti,ab.
65	1 or 2 or 3 or 4 or 5 or 6 or 7 or 8 or 9 or 10 or 11 or 12 or 13 or 14 or 15 or 16 or 17 or 18
66	19 or 20 or 21 or 22 or 23 or 24 or 25 or 26 or 27 or 28 or 29 or 30 or 31 or 32 or 34 or 35 or 36 or 41 or 42 or 43
67	44 or 45 or 46 or 47
68	48 or 49 or 50 or 51 or 52 or 53 or 54 or 55 or 56 or 57 or 58 or 59 or 60 or 61 or 62
69	65 and 66 and 67 and 68
70	limit 69 to yr="2010 -Current"

References:

1. Aiyegbusi OL, Kyte D, Cockwell P, et al. Measurement properties of patient-reported outcome measures (PROMs) used in adult patients with chronic kidney disease: a systematic review protocol. *BMJ Open* 2016;6(10):e012014. doi: 10.1136/bmjopen-2016-012014
