# Supplementary material for: Protocol for a scoping review exploring the use of patient-reported outcomes in adult social care
Source: BMJ Open. 2021 Apr 13;11(4):e045206. doi: 10.1136/bmjopen-2020-045206 (PMC8051391; doi:10.1136/bmjopen-2020-045206)
Supplement: Author's manuscript [file bmjopen-2020-045206.draft_revisions.pdf]

BMJ Open is committed to open peer review. As part of this commitment we make the peer review history of every article we publish publicly available.

When an article is published we post the peer reviewers' comments and the authors' responses online. We also post the versions of the paper that were used during peer review. These are the versions that the peer review comments apply to.

The versions of the paper that follow are the versions that were submitted during the peer review process. They are not the versions of record or the final published versions. They should not be cited or distributed as the published version of this manuscript.

BMJ Open is an open access journal and the full, final, typeset and author-corrected version of record of the manuscript is available on our site with no access controls, subscription charges or pay-per-view fees (http://bmjopen.bmj.com).

If you have any questions on BMJ Open's open peer review process please email info.bmjopen@bmj.com

BMJ Open

Protocol for a scoping review exploring the use of patient reported outcomes (PROs) in adult social care

Journal:	*BMJ Open*
Manuscript ID	bmjopen-2020-045206
Article Type:	Protocol
Date Submitted by the Author:	24-Sep-2020

Complete List of Authors:	Hughes, Sarah; University of Birmingham, Centre for Patient Reported Outcomes Research and Institute of Applied Health Research<br>Aiyegbusi, Olalekan ; University of Birmingham, Centre for Patient-Reported Outcomes Research; University of Birmingham, NIHR Birmingham Biomedical Research Centre, University of Birmingham and University Hospitals Birmingham NHS Foundation Trust<br>Lasserson, Daniel; University of Warwick Warwick Medical School<br>Collis, Philip; University of Birmingham, Centre for Patient Reported Outcomes Research and Institute of Applied Health Research<br>Cruz Rivera, Samantha; University of Birmingham, Centre for Patient Reported Outcomes Research and Institute of Applied Health Research<br>McMullan, Christel; University of Birmingham, Institute of Applied Health Research<br>Turner, Grace; University of Birmingham, Centre for Patient Reported Outcomes Research and Institute of Applied Health Research<br>Glasby, Jon; University of Birmingham, Department of Social Work and Social Care<br>Calvert , Melanie ; University of Birmingham, Centre for Patient Reported Outcomes Research and Institute of Applied Health Research

Keywords:	Quality in health care < HEALTH SERVICES ADMINISTRATION & MANAGEMENT, Health policy < HEALTH SERVICES ADMINISTRATION & MANAGEMENT, QUALITATIVE RESEARCH

SCHOLARONE™
Manuscripts

BMJ

I, the Submitting Author has the right to grant and does grant on behalf of all authors of the Work (as defined in the below author licence), an exclusive licence and/or a non-exclusive licence for contributions from authors who are: i) UK Crown employees; ii) where BMJ has agreed a CC-BY licence shall apply, and/or iii) in accordance with the terms applicable for US Federal Government officers or employees acting as part of their official duties; on a worldwide, perpetual, irrevocable, royalty-free basis to BMJ Publishing Group Ltd ("BMJ") its licensees and where the relevant Journal is co-owned by BMJ to the co-owners of the Journal, to publish the Work in this journal and any other BMJ products and to exploit all rights, as set out in our licence.

The Submitting Author accepts and understands that any supply made under these terms is made by BMJ to the Submitting Author unless you are acting as an employee on behalf of your employer or a postgraduate student of an affiliated institution which is paying any applicable article publishing charge ("APC") for Open Access articles. Where the Submitting Author wishes to make the Work available on an Open Access basis (and intends to pay the relevant APC), the terms of reuse of such Open Access shall be governed by a Creative Commons licence – details of these licences and which Creative Commons licence will apply to this Work are set out in our licence referred to above.

Other than as permitted in any relevant BMJ Author's Self Archiving Policies, I confirm this Work has not been accepted for publication elsewhere, is not being considered for publication elsewhere and does not duplicate material already published. I confirm all authors consent to publication of this Work and authorise the granting of this licence.

For peer review only

Protocol for a scoping review exploring the use of patient reported outcomes (PROs) in adult social care

Sarah E. Hughes[1,2]
Olalekan L. Aiyegbusi[1,2,3]
Dan Lasserson[2,4,5]
Philip Collis[1]
Samantha Cruz-Rivera[1,7]
Christel McMullan[1]
Grace Turner[1,3]
Jon Glasby[6]
Melanie Calvert[1,2,3,7]

[1]Centre for Patient Reported Outcome Research, Institute of Applied Health Research, University of Birmingham B15 2TT UK

[2]National Institute of Health Research (NIHR) Applied Research Centre (ARC) West Midlands, Institute of Applied Health Research, University of Birmingham B15 2TT UK

[3]NIHR Birmingham Biomedical Research Centre, NIHR Surgical Reconstruction and Microbiology Research Centre, University of Birmingham B15 2TT UK

[4]Warwick Medical School, University of Warwick, Coventry, CV4 7AL.

[5]Department of Acute Medicine, City Hospital, Sandwell and West Birmingham NHS Trust, Birmingham B18 7QH UK

[6]Department of Social Work and Social Care, University of Birmingham B15 2TT

[7]Birmingham Health Partners Centre for Regulatory Science and Innovation, University of Birmingham B15 2TT UK

Address for correspondence:
Sarah Hughes
Centre for Patient Reported Outcome Research (CPROR)
Institute of Applied Health Research, University of Birmingham
Edgbaston, Birmingham, UK
Tel: +44 121 414 3353
E-mail: s.e.hughes@bham.ac.uk

For peer review only

For peer review only - http://bmjopen.bmj.com/site/about/guidelines.xhtml

ABSTRACT

Introduction - Patient reported outcomes (PROs) are measures of a person's own views of their health, functioning, and quality of life. They are typically assessed using validated, self-completed questionnaires known as patient reported outcome measures (PROMs). PROs are widely used in healthcare settings to support care planning, clinical decision-making, facilitate communication between patients and healthcare providers, and enable service development and quality improvement. PROs have a potential role in the delivery of social care where people often have multiple and complex long term health conditions. However, the use of PROs in these contexts is currently unclear. The objective of this scoping review is to explore the evidence relating to the use of PROs in adult social care.

Methods and analyses - This scoping review will use the methodology proposed by the Joanna Briggs Institute, supported by the original scoping review framework proposed by Arksey and O'Malley. The electronic databases Medline (Ovid), PsychInfo (Ovid), ASSIA (ProQuest), Social Care Online (SCIE), Web of Science, and EMBASE (Ovid) will be searched together with a grey literature search and hand searching of citations and reference lists to identify eligible studies, reports, and other publically available documents published since 2010. No restrictions on study design or language of publication will be applied. Screening and data extraction will be completed independently by two reviewers, with discrepancies resolved through discussion, involving a third reviewer as required. Quality appraisal of the included documents will use the CASP and AACODS checklists. A customised data charting table will be used for data extraction, with analysis of qualitative data using the Framework Method. The review findings will be presented as tables and in a narrative summary.

Ethics and dissemination - Ethical review is not required as scoping reviews are a form of secondary data analysis that synthesise data from publically available sources. Review findings will be shared with service users and other relevant stakeholders. Findings will also be disseminated through a peer-reviewed publication and conference presentations. This protocol is registered on the Open Science Framework (www.osf.io).

For peer review only

For peer review only - http://bmjopen.bmj.com/site/about/guidelines.xhtml

STRENGTHS AND LIMITATIONS

- This scoping review will follow robust and established guidelines for the conduct of scoping reviews, ensuring transparency and rigour.
- Service user and public involvement in the design and conduct of the review and the planned consultation exercise (protocol reported elsewhere) will ensure the findings are supported by stakeholder experiences, further helping to identify evidence gaps and research priorities.
- An appraisal of the methodological quality of studies is not a requirement of a scoping review. However, a quality appraisal of the included research articles will be conducted to gather insights into the types, sources, and quality of the evidence around the use of PROs in adult social care to support recommendations for future research.

For peer review only

For peer review only - http://bmjopen.bmj.com/site/about/guidelines.xhtml

INTRODUCTION

People with care and support needs often live with multiple and complex, long term health conditions.[1] They are likely to experience greater levels of disability and have complex care needs.[2 3] An understanding of a person's own views about their health can help ensure care is provided in a way that is holistic, person-centred, and responsive. Such insights are particularly relevant in the UK where a fragmented health and social care system makes the coordination of care challenging.[4]

Individuals' perspectives about their health are most useful to care providers when these first-hand accounts are both relevant and reliably measured.[5] A patient reported outcome (PRO) is "a measurement of any aspect of a patient's health that comes directly from the patient without the interpretation of the patient's responses by anyone else".[6] PROs include symptom severity of the impact of a disease or its treatment on a patient's health-related quality of life (including functioning and social and emotional well-being).[7] PROs are typically measured using self-report questionnaires known as patient reported outcome measures (PROMs).

PROs are implemented widely across different levels of health care.[8] At an individual level, PROs may be used to inform decision-making around treatment, monitor disease symptoms and treatment adherence, and facilitate communication between the patient and their clinical team. At a systems level, PROs may be used support benchmarking including service evaluation and commissioning of services, inform policy-making, and support quality improvement initiatives. PROs are used as endpoints within clinical trials, support regulatory approval of new medicinal products and devices, and inform clinical guidelines and health policy.[9-11] PROs are valued due their capability to ensure the patient's unique perspective is represented in the measurement of health and when capturing the effectiveness of healthcare interventions.

Despite their established use in healthcare, the use of PROs in social care is less clear. A better understanding of how PROs are utilised, by whom, and the barriers and facilitators to their utilisation could help to inform initiatives designed to improve the delivery of social care. A better understanding of the use of PROs in social care, and particularly in the delivery of integrated health and social care, may be considered essential as decision-making around care becomes increasingly collaborative, person-focussed, and data driven.[12] Clarification on PRO use is especially relevant in light of the Covid-19 pandemic which has resulted in the increased use of telemedicine, virtual consultations, and remote monitoring.[13 14] PROs stand to play an integral role in ensuring that people with care and support needs have their views about their health captured in a rigorous and standardised manner.[15]

For peer review only - http://bmjopen.bmj.com/site/about/guidelines.xhtml

Published reviews often study PROs and PROMs within a specific context, health condition, or population. For example, reviews have explored the use of PROs in primary care and investigated stakeholders' perspectives regarding the use of PROMs with frail, older adults living at home.[16 17] Reviews have also been conducted that focus on the use of PROMs in end-of-life care and in the care of people with dementia and other long term conditions.[18-21] A review focussing on the use of PROs in the broader context of social care has not been identified.

Consistent with the remit of scoping reviews as a form of knowledge synthesis, the aim of this scoping review is to explore the existing evidence to understand the current state of knowledge on the use of PROs in adult social care. To achieve this aim, the specific review objectives are: 1) to identify PROs used in social care interventions; 2) to characterise how PROs and PROMs (as the instruments used to measure PROs), have been applied in these contexts; and to chart the evidence relating to 3) their effectiveness; 4) barriers and facilitators to their implementation; and 5) stakeholder views on the use of PROs in social care. A sub analysis to explore the use of PROs in the delivery of integrated health and social care, as a special case, will also be undertaken.[22]

A shared language for PROs

The term "patient-reported outcome" is a reflection of PROs' origins in medicine and healthcare. However, in the context of social care people with care and support needs rarely identify as patients, preferring to identify as service users or citizens.[23] In this review on the use of PROs within adult social care, the term PRO will refer to a "patient or *person-reported* outcome" whilst retaining the United States Food and Drug Administration's (FDA) definition of a PRO as a measure of health status.[6 11 24] Therefore, in this review, a "patient or person-reported outcome" (PRO) will refer specifically to health-related outcomes reported directly by an individual (i.e., disease symptoms, treatment adherence, health-related quality of life) and exclude measurements of a person's experience of care or social care-related outcomes.

METHODS AND ANALYSIS

Protocol design, registration, and reporting

Scoping reviews are conducted to understand the existing research on a given topic and often serve as a precursor to systematic reviews.[25] Specifically, scoping reviews seek to identify and map the available evidence and, in so doing, identify knowledge gaps and directions for

For peer review only - http://bmjopen.bmj.com/site/about/guidelines.xhtml

future research.[26] Frequently, scoping reviews are complemented by stakeholder consultation exercises to ensure a holistic understanding of the topic that is informed by both the published evidence and lived experience.[27] This scoping review will use the methodology proposed by the Joanna Briggs Institute, supported by the original scoping framework proposed by Arksey and O'Malley.[27 28] The Preferred Reporting Items for Systematic Reviews and Meta-analyses guidance for protocol development (PRISMA-P) and scoping reviews (PRISMA-ScR) and JBI reporting checklists were consulted when preparing this protocol.[28-30] This review protocol is registered online with the Open Science Framework (www.osf.io).

Eligibility criteria

Participants

The review will consider sources of evidence that report on adults (≥ 18 years of age) who are users of social care services or receive integrated health and social care. No limitations with regard to an individual's health condition(s) will be applied.

Concept

Sources of evidence that provide information on the use of PROs and PROMs in integrated and/or social care will be considered for inclusion in the review.

Context

Sources of evidence reporting on a social care or integrated care intervention will be considered. The review will exclude studies and other evidence sources set in primary and secondary care context if there is no mention of an integrated or social care intervention.

Types of sources

This scoping review will consider all sources of published evidence on the use of PROs and PROMs in adult social care. Sources of evidence will include primary research studies, websites, guidelines, government reports, and policy-documents. Expert opinions, editorials, and studies reporting secondary data analysis (i.e., systematic reviews) will also be included.

Information sources

The searches will be conducted in three stages: 1) electronic database searching; 2) grey literature searching; and 3) hand searching the reference lists and citations of included sources to identify further studies for inclusion. Electronic database selection was based on recommendations for optimal database searching for sensitivity.[31]

For peer review only - http://bmjopen.bmj.com/site/about/guidelines.xhtml

The electronic databases Medline (Ovid), Embase (Ovid), PsychInfo (Ovid), HMIC (Ovid), Social Care Online (SCIE), ASSIA (Pro Quest) and Web of Science (Pro Quest) and the grey literature database Open Grey and Google will be searched for the period 2010 to present date, with no language limitation. Sources in languages other than English will be translated. Grey literature will be defined as "work that is produced on all levels of government, academia, business, and industry in print and electronic formats but which is not controlled by commercial publishers".[32] The use of PROMs has increased substantially over the last decade, commensurate with introduction of the UK National PROMs programme, publication of the FDA's guidance on PROMs, and the UK government's NHS white paper outlining government's vision for a patient-centred NHS and greater integration of health and social care.[6 33 34] As such, a ten year date limit on searches was considered appropriate for locating relevant sources on the current application of PROs in adult social care.

Search strategy

The Medline search strategy will be developed first and then adapted for use with other databases. The search strategy will combine MeSH terms (and relevant synonyms) and the Boolean operators "AND" and "OR" for search strings relating to: 1) the concept of interest (i.e., PROs and PROMs); 2) the population of adults who use social care and/or integrated care services; and contextual factors including 3) type of integrated care/social care intervention and 4) PRO implementation (e.g., barriers and facilitators). Terms will be developed by the review team with support from an information science specialist (SB). Service user partner and co-investigator (PC) will be consulted to identify specific search terms related to integrated care and social care. The grey literature search will take place after searches of the electronic databases have been completed to enable to study team to immerse themselves in the literature and come to better understand the evidence base. Hand searching of citations and reference lists of included documents will be undertaken at each stage of the search process. The Medline search strategy is presented in Supplementary file X.

Selection of sources of evidence

A team of reviewers (SH, GT, CM, SCR) will conduct title/abstract and full-text screening of all records against pre-specified eligibility criteria to ascertain suitability for inclusion in the review. All records will be screened by a minimum of two reviewers independently. Disagreements between reviewers will be resolved through discussion. If necessary, a third reviewer will be involved if consensus cannot be reached. The eligibility

For peer review only - http://bmjopen.bmj.com/site/about/guidelines.xhtml

criteria will be pre-tested on a sample of abstracts to ensure that evidence sources discussing the use of PROs and PROMs in integrated/social care are captured. Records identified from electronic databases, the grey literature, and hand-searching will be imported to Endnote 9.3.3 (www.endnote.com) reference management software and duplicate records will be removed. All screening will be conducted within Endnote. Any retrieved documents deemed to meet the inclusion criteria will go forward for full-text review. For articles excluded during full text screening, a reason for exclusion will be documented. Disagreements on article inclusion will be resolved through discussion by members of the review team until consensus is achieved. The study selection process will be documented in a PRISMA flow diagram.

Critical appraisal of individual sources of evidence

Assessment of the methodological quality of included studies is not a requirement of scoping reviews.[28] The decision to undertake a quality appraisal will be governed by the number and type of sources identified by the searches. If feasible, a formal quality assessment will be undertaken using the Critical Appraisal Skills Programme (CASP) checklists and the AACODS checklist for quality appraisal of the grey literature.[35 36] Appraisals will be undertaken to characterise the overall quality of the evidence base and no study will be excluded on the basis of its methodological quality. Two reviewers will independently appraise each study for quality. Findings will be collated by study type and presented in tabular format along with a textual description of findings. To ensure transparency of reporting, interrater agreement and reliability will be determined by calculating an intra-class coefficient (ICC) statistic, Cohen's kappa (κ), and percentage agreement.[37 38]

Data charting process

Data extraction, termed data charting in scoping reviews, aims to provide a "logical and descriptive summary of results that aligns with the review questions".[28] A standardised template will be developed in Excel and piloted prior to commencing data charting. Key characteristics of the data source and information relevant to answering the review questions will be extracted independently by two reviewers with discrepancies resolved through discussion. As a scoping review is an iterative process, the data extraction form will be refined and updated throughout the course of the review and in response to the emergent findings.

Data items

For peer review only - http://bmjopen.bmj.com/site/about/guidelines.xhtml

The data items to be extracted for each data source comprise the publication title, date of publication, authors, country (location of study), study aims and objectives, study design, study setting, integrated/social care intervention, study population, study data collection method, data analysis method, consumer and public involvement in the included studies, study outcome(s), PRO, the PROM intervention, method of PROM administration, context of use, frequency/time points of administration, PRO data management, PROM administrator (state occupation), individual-level outcomes in response to the PROM intervention, process and system-level outcomes in response to the PROM intervention, and barriers/enablers to implementation. Information specific to each PROM will be charted. Data items will include a list of studies using each PROM and, if available, a description of the PROMs' characteristics, including target population, construct domains, number of items and number of subscales, mode of administration, whether the PROM is patient or proxy-administered, recall period, response options, range of scores, scoring, language of the original publication and available translations.

Data summary

A qualitative content analysis in scoping reviews is generally descriptive in nature.[28] Following data charting, findings from the included sources will be collated with analysis of common themes relating to the aims of the review. Given the anticipated diversity of the included data sources, framework analysis, conducted using NVivo (Version 1.3) qualitative data analysis software, will be used together with data charting to capture key themes.[39] These results will be presented using tables and diagrams accompanied by a narrative summary.[28]

Piloting/Calibration exercise

All phases of the scoping review will be pretested. The JBI recommended procedure for pilot testing will be used.[28] For screening, the titles and abstracts of 25 randomly selected articles will be screened by the review team, applying the *a priori* review eligibility criteria. Screening will commence once inter-rater agreement of 75% or greater is achieved. Inter-rater agreement that is less than 75% will prompt the study team to review and refine the inclusion criteria. This process of pilot testing will continue iteratively until the 75% agreement threshold is met.

For peer review only - http://bmjopen.bmj.com/site/about/guidelines.xhtml

The data charting template will also be piloted by two reviewers to ensure the capture of data relevant to the aims and objective of the review. As the review progresses, the reviewers may wish to extract additional information of relevance to the review objectives; therefore, further revisions to the data chart template may be made iteratively as the review progresses.[28]

Changes to the protocol

The exploratory nature and breadth of a scoping review mean that changes to the protocol may be required as new information becomes available and as the review progresses. Any protocol deviations or refinements, together with a rationale for these changes, will be documented clearly in the review report.

Service user and public involvement

This scoping review was conceived and developed with service user and public involvement (PC). As co-investigator, PC will contribute to the development of the search strategies, the study protocol, consensus discussions, will review all study-related documents, and co-develop a lay summary to facilitate dissemination of the review findings. In addition, this work will be discussed with the NIHR Long Term Conditions Theme 1 Patient and Public Involvement (PPI) Stakeholder Advisory Group at various points in the review process. In the reporting of this review the Guidance for Reporting Involvement of Patients and the Public-2 short form (GRIPP-2SF) checklist will be used to document service user and public involvement.[40]

Consultations

Stakeholder consultation is recommended practice in the conduct of scoping reviews.[27] A qualitative mixed-methods study to explore stakeholder perspectives on the use of PROs in adult social care and integrated care will be undertaken separately to extend and validate findings from this scoping review. The planned stakeholder consultation aims to provide insights regarding PRO and PROM use beyond what is reported in published sources. Consistent with principles of person-centred research, this study will be co-produced with people who use health and social care services.

DISCUSSION

PROs can provide key data that enable health and social care practitioners to better understand a person's views about their health, allowing them to provide effective, efficient,

For peer review only - http://bmjopen.bmj.com/site/about/guidelines.xhtml

and compassionate care. This scoping review will be the first to provide an overview of the evidence around the use of PROs with adults who use social care services. Given the disparate nature of the evidence, the use of a scoping review methodology is appropriate as it enables the review to evolve in an iterative manner that is responsive to the evidence.

REFERENCES

1. Goodwin N, Curry N, Naylor C, et al. Managing People with Long Term Conditions. London: The King's Fund 2010.
2. Vogeli C, Shields AE, Lee TA, et al. Multiple Chronic Conditions: Prevalence, Health Consequences, and Implications for Quality, Care Management, and Costs. *J Gen Intern Med* 2007;22(3):391-95. doi: 10.1007/s11606-007-0322-1
3. Barnett K, Mercer SW, Norbury M, et al. Epidemiology of multimorbidity and implications for health care, research, and medical education: a cross-sectional study. *Lancet* 2012;380(9836):37-43. doi: 10.1016/S0140-6736(12)60240-2
4. Glasby J. The holy grail of health and social care integration. *BMJ* 2017;356:j801. doi: 10.1136/bmj.j801
5. FDA. Principles for Selecting, Developing, Modifying, and Adapting PatientReported Outcome Instruments for Use in Medical Device Evaluation: Draft Guidance for Industry and Food and Drug Administration Staff, and Other Stakeholders. 2020. (accessed 31 August 2020).
6. FDA. Guidance for industry: Patient-reported outcome measures: Use in medicinal product development to support labelling claims, 2009.
7. Wilson IB. Linking clinical variables with health-related quality of life. A conceptual model of patient outcomes. *JAMA* 1995;273(1):59-65. doi: 10.1001/jama.273.1.59
8. Calvert M, Kyte D, Price G, et al. Maximising the impact of patient reported outcome assessment for patients and society. *BMJ* 2019;364:k5267. doi: 10.1136/bmj.k5267
9. Kyte D, Duffy H, Fletcher B, et al. Systematic evaluation of the patient-reported outcome (PRO) content of clinical trial protocols. *PloS One* 2014;9(10):e110229-e29. doi: 10.1371/journal.pone.0110229
10. FDA. FDA Patient-Focused Drug Development Guidance Series for Enhancing the Incorporation of the Patient's Voice in Medical Product Development and Regulatory Decision Making 2020 [updated 18/06/202020 September 2020]. Available from: https://www.fda.gov/drugs/development-approval-process-drugs/fda-patient-focused-drug-development-guidance-series-enhancing-incorporation-patients-voice-medical accessed 20 September 2020.
11.FDA. Principles for Selecting, Developing, Modifying, and Adapting Patient-reported Outcome Instruments for Use in Medical Device Evaluation: Draft Guidance for Industry and Food and Drug Administration Staff, and Other Stakeholders: US Food and Drug Administration 2020.
12. Fund TKs. How to develop evidence-based solutions to improve patient journeys: The King's Fund, 2020.
13. Greenhalgh T, Koh GCH, Car J. Covid-19: a remote assessment in primary care. *BMJ* 2020;368:m1182. doi: 10.1136/bmj.m1182
14. Aiyegbusi OL, Calvert MJ. Patient-reported outcomes: central to the management of COVID-19. *Lancet* 2020;396(10250):531. doi: 10.1016/s0140-6736(20)31724-4

For peer review only - http://bmjopen.bmj.com/site/about/guidelines.xhtml

15. Taylor PC. Adopting PROs in virtual and outpatient management of RA. *Nat Rev Rheumatol* 2020;16(9):477-78. doi: 10.1038/s41584-020-0449-6
16. Murphy M, Hollinghurst S, Salisbury C. Identification, description and appraisal of generic PROMs for primary care: a systematic review. *BMC Fam Pract* 2018;19(1):41. doi: 10.1186/s12875-018-0722-9
17. Schick-Makaroff K, Karimi-Dehkordi M, Cuthbertson L, et al. Using Patient- and Family-Reported Outcome and Experience Measures Across Transitions of Care for Frail Older Adults Living at Home: A Meta-Narrative Synthesis. *Gerontologist* 2020 doi: 10.1093/geront/gnz162
18. Kearns T, Cornally N, Molloy W. Patient reported outcome measures of quality of end-of-life care: A systematic review. *Maturitas* 2017;96:16-25. doi: 10.1016/j.maturitas.2016.11.004
19. Johnston BM, Flemming K, Narayanasamy MJ, et al. Patient reported outcome measures for measuring dignity in palliative and end of life care: a scoping review. *BMC Health Serv Res* 2017;17
20. Ayton DR, Gardam ML, Pritchard EK, et al. Patient-Reported Outcome Measures to Inform Care of People With Dementia—A Systematic Scoping Review. *Gerontologist* 2020 doi: 10.1093/geront/gnz179
21. Forestier B, Anthoine E, Reguiai Z, et al. A systematic review of dimensions evaluating patient experience in chronic illness. *Health Qual Life Outcomes* 2019;17(1):19-19. doi: 10.1186/s12955-019-1084-2
22. Humphries R. Integrated health and social care in England – Progress and prospects. *Health Policy* 2015;119(7):856-59. doi: https://doi.org/10.1016/j.healthpol.2015.04.010
23. Glasby J. Understanding health and social care (third edition). REV - Revised, 3 ed: Bristol University Press 2017.
24. Klose K, Kreimeier S, Tangermann U, et al. Patient- and person-reports on healthcare: preferences, outcomes, experiences, and satisfaction - an essay. *Health Econ Rev* 2016;6(1):18-18. doi: 10.1186/s13561-016-0094-6
25. Peterson J, Pearce PF, Ferguson LA, et al. Understanding scoping reviews: Definition, purpose, and process. *J Am Assoc Nurse Pract* 2017;29(1)
26. Munn Z, Peters MDJ, Stern C, et al. Systematic review or scoping review? Guidance for authors when choosing between a systematic or scoping review approach. *BMC Medl Res Methodol* 2018;18(1):143. doi: 10.1186/s12874-018-0611-x
27. Arksey H, O'Malley L. Scoping studies: towards a methodological framework. *International J Soc Res Methodol* 2005;8(1):19-32. doi: 10.1080/1364557032000119616
28. Peters M, Godfrey C, McInerney P, et al. Chapter 11: Scoping Reviews (2020 Version). In: Aromataris E, Munn Z, eds. JBI Manual for Evidence Synthesis: JBI 2020.
29. Moher D, Shamseer L, Clarke M, et al. Preferred reporting items for systematic review and meta-analysis protocols (PRISMA-P) 2015 statement. *Syst Rev* 2015;4(1):1. doi: 10.1186/2046-4053-4-1
30. Tricco AC, Lillie E, Zarin W, et al. PRISMA Extension for Scoping Reviews (PRISMA-ScR): Checklist and Explanation. *Ann Intern Med* 2018;169(7):467-73. doi: 10.7326/m18-0850 [published Online First: 2018/09/05]
31. Bramer WM, Rethlefsen ML, Kleijnen J, et al. Optimal database combinations for literature searches in systematic reviews: a prospective exploratory study. *Syst Rev* 2017;6(1):245. doi: 10.1186/s13643-017-0644-y

For peer review only

For peer review only - http://bmjopen.bmj.com/site/about/guidelines.xhtml

32. Godin K, Stapleton J, Kirkpatrick SI, et al. Applying systematic review search methods to the grey literature: a case study examining guidelines for school-based breakfast programs in Canada. *Syst Rev* 2015;4(1):138. doi: 10.1186/s13643-015-0125-0
33. Devlin NJ, Appleby J. Getting the Most Out of PROMs. London: The King's Fund, 2010.
34. Equity and Excellence: Liberating the NHS. In: Health Do, ed. London: HMSO, 2010.
35. CASP Checklists 2020 [Available from: https://casp-uk.net/casp-tools-checklists/ accessed 28 August 2020.
36. Tyndall J. ACCODS checklist for appraising grey literature. 2010. https://dspace.flinders.edu.au/jspui/bitstream/2328/3326/4/AACODS_Checklist.pdf (accessed 20 September 2020).
37. Shrout PE, Fleiss JL. Intraclass correlations: uses in assessing rater reliability. *Psychol Bull* 1979;86(2):420-8. doi: 10.1037//0033-2909.86.2.420 [published Online First: 1979/03/01]
38. Cohen J. A coefficient of agreement for nominal scales. *Educ Psychol Meas* 1960;20:37-46. doi: 10.1177/001316446002000104
39. Gale NK, Heath G, Cameron E, et al. Using the framework method for the analysis of qualitative data in multi-disciplinary health research. *BMC Med Res Methodol* 2013;13:117. doi: 10.1186/1471-2288-13-117
40. Staniszewska S, Brett J, Simera I, et al. GRIPP2 reporting checklists: tools to improve reporting of patient and public involvement in research. *Res Involv Engagem* 2017;3(1):13. doi: 10.1186/s40900-017-0062-2

Acknowledgments

The authors wish to thank Sue Bayliss, Information Specialist, University of Birmingham for her assistance in developing the search strategy.

Contributors

SH, MC, OLA, DL conceptualised the study and contributed to the study design. SH and PC developed the search strategy. SH drafted and edited the manuscript with all authors providing critical revisions to the manuscript and supplementary material. The final version was read and approved by all authors.

Funding

This research is funded by the National Institute for Health Research (NIHR) Applied Research Collaboration (ARC) West Midlands. The views expressed are those of the author(s) and not necessarily those of the NIHR or the Department of Health and Social Care.

Competing interests

SH is supported by the National Institute of Health Research (NIHR) Applied Research Centre (ARC), West Midlands. SH is also company director of Narra Consulting Limited and undertakes consultancy work for Cochlear Ltd. MC is a National Institute for Health Research (NIHR) Senior Investigator and receives funding from the National Institute for Health Research (NIHR) Birmingham Biomedical Research Centre, the NIHR Surgical Reconstruction and Microbiology Research Centre and NIHR ARC West Midlands at the at the University of Birmingham and University Hospitals Birmingham NHS Foundation Trust, Health Data Research UK, Innovate UK (part of UK Research and Innovation), Macmillan Cancer Support, UCB Pharma MC has received personal fees from Astellas, Takeda, Merck,

For peer review only - http://bmjopen.bmj.com/site/about/guidelines.xhtml

Daiichi Sankyo, Glaukos, GSK and the Patient-Centered Outcomes Research Institute (PCORI) outside the submitted work. OLA is supported by the National Institute of Health Research (NIHR) Birmingham Biomedical Research Centre (BRC), Birmingham, West Midlands. OLA also receives funding from the Health Foundation and has received personal fees from Gilead Sciences. DL is supported by the NIHR ARC West Midlands and the NIHR Community Healthcare MedTech and IVD Cooperative, hosted by Oxford Health NHS Foundation Trust. JG is a non-executive director of University Hospitals Birmingham, adjunct professor at Curtin University, member of the leadership team for an ESRC large grant on sustainable adult social care, and member of the expert advisory group for West Midlands ARC. PC, GT, SCR, and CM have no conflicts of interest to declare.

Provenance and peer review

Not commissioned; externally peer reviewed.

Patient consent for publication

Not required

Data sharing statement

No additional data are available.

Open access

This is an Open Access article distributed in accordance with the Creative Commons Attribution Non Commercial (CC-BY-NC 4.0) license, which permits others to distribute, remix, adapt, build upon this work non-commercially, and license their derivative works on different terms, provided the original work is properly cited and the use is non-commercial. See: http://creativecommons.org/licenses/by-nc/4.0/.

For peer review only

Supplemental File 1 – Search strategy

Note: Lines 48-72 are based on the modified Oxford PROM filter and the search strategy developed by Aiyegbusi et al.[1]

	**Setting**
1	respite care/
2	respite care.ti,ab.
3	hospice/ or home care/ or palliative care/ or terminally ill patients/
4	exp client transfer/ or exp facility discharge/ or exp hospital discharge/ or exp patient selection/ or exp professional referral/ or exp psychiatric hospital discharge/ or exp treatment refusal/ or exp treatment termination/
5	patient transfer.ti,ab.
6	hospice.ti,ab.
7	residential home.ti,ab.
8	care home*.ti,ab.
9	aged care home*.ti,ab.
10	nursing homes/
11	retirement home*.ti,ab.
12	adult day care centre*.ti,ab.
13	homes for the aged.ti,ab.
14	self-care skills/ or aging in place/ or independent living programs/ or rehabilitation/
15	independent living.ti,ab.
16	personalised budget*.ti,ab.
17	reablement.ti,ab.
18	home nursing.ti,ab.
	**Population**
19	aged.ti,ab.
20	geriatric patients/
21	old* person.ti,ab.
22	(elderly or geriatric).ti,ab.
23	exp Disabilities/ or exp Intellectual Development Disorder/ or exp Developmental Disabilities/
24	disabled person*.ti,ab.
25	disabled people.ti,ab.
26	(disabled adj3 person*).ti,ab.
27	vulnerable population*.ti,ab.
28	vulnerable person*.ti,ab.
29	intellectual development disorder/
30	exp Autism Spectrum Disorders/
31	autism spectrum disorder.ti,ab.
32	intellectual disabilit*.ti,ab.
33	learning disabilit*.ti,ab.
34	exp Chronic Illness/ or exp Long Term Care/

For peer review only

35	long term condition*.ti,ab.
36	chronic illness.ti,ab.
37	exp substance-related disorders/
38	mental* ill*.ti,ab.
39	drug user.ti,ab.
40	homeless.ti,ab.
41	clients/
42	service user*.ti,ab.
43	adult.ti,ab.
	**Type of Care**
44	exp Social Services/
45	social care.ti,ab.
46	integrated services/
47	integrated care.ti,ab.
	**PROs**
48	(PRO integration or Clinical PRO application* or telePRO or automated PRO algorithm* or screening purpose* or PRO questionnaire* or Patient-reported outcome questionnaire* or PROM or Patient-reported outcome measure* or Patient reported outcome measure* or Patient-reported symptom* or Patient-centred care or Patient self-report* or Self-report health or Self-rated health or Self-reported measure* of health or Health outcome* or Health communication* or Hospital performance evaluation* or Automated telephone survey system* or paper-based survey* or web-based survey* or web-based PRO platform* or web-based system* or PRO collection* or PRO measure* or PRO intervention* or PRO assessment intervention* or PRO data or PRO assessment* or Routine PRO assessment* or Routine PRO collection or Symptom assessment* or Symptom monitoring or Symptom data or Functional status or Electronic PRO assessment* or Electronic PRO system* or ePRO or ePRO* or ePRO system* or PRO system* or Generic PRO system* or PRO-based clinical alert system*).ti,ab.
49	exp Patient Reported Outcome Measures/
50	((((patient adj reported adj outcome adj measure*) or patient) adj reported adj outcome*) or capability or capabilities).ti,ab.
51	((disability or function or functional or functions or subjective or utility or utilities or wellbeing or well being) adj2 (index or indices or instrument or instruments or measure or measures or questionnaire* or profile or profiles or scale or scales or score or scores or status or survey or surveys)).ti,ab.
52	((patient or self or carer or proxy) adj (appraisal* or appraised or report or reported or reporting or rated or rating or based or assessed or assessment*)).ti,ab.
53	self-report/
54	health status/
55	(health index* or health indices or health status or health profile*).ti,ab.
56	exp "Quality of Life"/
57	quality of life.ti,ab.
58	(HR-PRO or HRPRO or HRQL or HRQoL or QL or QoL).ti,ab.

For peer review only

59	(Patient Centred Care or person centred care or person centred coordinated care or p3c or person centred outcome*).ti,ab. or person reported outcome*.mp. or individualised patient reported outcome measur*.ti,ab. or iprom.ti,ab.
60	patient centered care.ti,ab.
61	person centered care.ti,ab.
62	(Patient Centered Care or person centered care or person centered coordinated care or p3c or person centred outcome*).ti,ab. or person reported outcome*.mp. or individualised patient reported outcome measur*.ti,ab. or iprom.ti,ab.
	**Implementation**
63	((implementation or implementation science).ti,ab. or implementation.mp.) adj3 science.ti,ab.
64	"barriers and facilitators".ti,ab.
65	1 or 2 or 3 or 4 or 5 or 6 or 7 or 8 or 9 or 10 or 11 or 12 or 13 or 14 or 15 or 16 or 17 or 18
66	19 or 20 or 21 or 22 or 23 or 24 or 25 or 26 or 27 or 28 or 29 or 30 or 31 or 32 or 34 or 35 or 36 or 41 or 42 or 43
67	44 or 45 or 46 or 47
68	48 or 49 or 50 or 51 or 52 or 53 or 54 or 55 or 56 or 57 or 58 or 59 or 60 or 61 or 62
69	65 and 66 and 67 and 68
70	limit 69 to yr="2010 -Current"

References:

1. Aiyegbusi OL, Kyte D, Cockwell P, et al. Measurement properties of patient-reported outcome measures (PROMs) used in adult patients with chronic kidney disease: a systematic review protocol. *BMJ Open* 2016;6(10):e012014. doi: 10.1136/bmjopen-2016-012014

For peer review only

Preferred Reporting Items for Systematic reviews and Meta-Analyses extension for Scoping Reviews (PRISMA-ScR) Checklist

SECTION	ITEM	PRISMA-ScR CHECKLIST ITEM	REPORTED ON PAGE #
**TITLE**			
Title	1	Identify the report as a scoping review.	1
**ABSTRACT**			
Structured summary	2	Provide a structured summary that includes (as applicable): background, objectives, eligibility criteria, sources of evidence, charting methods, results, and conclusions that relate to the review questions and objectives.	2
**INTRODUCTION**			
Rationale	3	Describe the rationale for the review in the context of what is already known. Explain why the review questions/objectives lend themselves to a scoping review approach.	3-4
Objectives	4	Provide an explicit statement of the questions and objectives being addressed with reference to their key elements (e.g., population or participants, concepts, and context) or other relevant key elements used to conceptualize the review questions and/or objectives.	4
**METHODS**			
Protocol and registration	5	Indicate whether a review protocol exists; state if and where it can be accessed (e.g., a Web address); and if available, provide registration information, including the registration number.	5
Eligibility criteria	6	Specify characteristics of the sources of evidence used as eligibility criteria (e.g., years considered, language, and publication status), and provide a rationale.	5
Information sources*	7	Describe all information sources in the search (e.g., databases with dates of coverage and contact with authors to identify additional sources), as well as the date the most recent search was executed.	5-6
Search	8	Present the full electronic search strategy for at least 1 database, including any limits used, such that it could be repeated.	6 + Appendix
Selection of sources of evidence†	9	State the process for selecting sources of evidence (i.e., screening and eligibility) included in the scoping review.	6-7
Data charting process‡	10	Describe the methods of charting data from the included sources of evidence (e.g., calibrated forms or forms that have been tested by the team before their use, and whether data charting was done independently or in duplicate) and any processes for obtaining and confirming data from investigators.	7
Data items	11	List and define all variables for which data were sought and any assumptions and simplifications made.	7-8
Critical appraisal of individual sources of evidence§	12	If done, provide a rationale for conducting a critical appraisal of included sources of evidence; describe the methods used and how this information was used in any data synthesis (if appropriate).	7

For peer review only

SECTION	ITEM	PRISMA-ScR CHECKLIST ITEM	REPORTED ON PAGE #
Synthesis of results	13	Describe the methods of handling and summarizing the data that were charted.	8
**RESULTS**			
Selection of sources of evidence	14	Give numbers of sources of evidence screened, assessed for eligibility, and included in the review, with reasons for exclusions at each stage, ideally using a flow diagram.	N/A
Characteristics of sources of evidence	15	For each source of evidence, present characteristics for which data were charted and provide the citations.	N/A
Critical appraisal within sources of evidence	16	If done, present data on critical appraisal of included sources of evidence (see item 12).	N/A
Results of individual sources of evidence	17	For each included source of evidence, present the relevant data that were charted that relate to the review questions and objectives.	N/A
Synthesis of results	18	Summarize and/or present the charting results as they relate to the review questions and objectives.	N/A
**DISCUSSION**			
Summary of evidence	19	Summarize the main results (including an overview of concepts, themes, and types of evidence available), link to the review questions and objectives, and consider the relevance to key groups.	N/A
Limitations	20	Discuss the limitations of the scoping review process.	10
Conclusions	21	Provide a general interpretation of the results with respect to the review questions and objectives, as well as potential implications and/or next steps.	N/A
**FUNDING**			
Funding	22	Describe sources of funding for the included sources of evidence, as well as sources of funding for the scoping review. Describe the role of the funders of the scoping review.	12

JBI = Joanna Briggs Institute; PRISMA-ScR = Preferred Reporting Items for Systematic reviews and Meta-Analyses extension for Scoping Reviews.
* Where *sources of evidence* (see second footnote) are compiled from, such as bibliographic databases, social media platforms, and Web sites.
† A more inclusive/heterogeneous term used to account for the different types of evidence or data sources (e.g., quantitative and/or qualitative research, expert opinion, and policy documents) that may be eligible in a scoping review as opposed to only studies. This is not to be confused with *information sources* (see first footnote).
‡ The frameworks by Arksey and O'Malley (6) and Levac and colleagues (7) and the JBI guidance (4, 5) refer to the process of data extraction in a scoping review as data charting.
§ The process of systematically examining research evidence to assess its validity, results, and relevance before using it to inform a decision. This term is used for items 12 and 19 instead of "risk of bias" (which is more applicable to systematic reviews of interventions) to include and acknowledge the various sources of evidence that may be used in a scoping review (e.g., quantitative and/or qualitative research, expert opinion, and policy document).

From: Tricco AC, Lillie E, Zarin W, O'Brien KK, Colquhoun H, Levac D, et al. PRISMA Extension for Scoping Reviews (PRISMAScR): Checklist and Explanation. Ann Intern Med. 2018;169:467–473. doi: 10.7326/M18-0850.

For peer review only - http://bmjopen.bmj.com/site/about/guidelines.xhtml

BMJ Open

Protocol for a scoping review exploring the use of patient reported outcomes in adult social care

Journal:	*BMJ Open*
Manuscript ID	bmjopen-2020-045206.R1
Article Type:	Protocol
Date Submitted by the Author:	02-Feb-2021

Complete List of Authors:	Hughes, Sarah; University of Birmingham, Centre for Patient Reported Outcomes Research and Institute of Applied Health Research<br>Aiyegbusi, Olalekan ; University of Birmingham, Centre for Patient-Reported Outcomes Research; University of Birmingham, NIHR Birmingham Biomedical Research Centre, University of Birmingham and University Hospitals Birmingham NHS Foundation Trust<br>Lasserson, Daniel; University of Warwick Warwick Medical School<br>Collis, Philip; University of Birmingham, Centre for Patient Reported Outcomes Research and Institute of Applied Health Research<br>Cruz Rivera, Samantha; University of Birmingham, Centre for Patient Reported Outcomes Research and Institute of Applied Health Research<br>McMullan, Christel; University of Birmingham, Institute of Applied Health Research<br>Turner, Grace; University of Birmingham, Centre for Patient Reported Outcomes Research and Institute of Applied Health Research<br>Glasby, Jon; University of Birmingham, Department of Social Work and Social Care<br>Calvert , Melanie ; University of Birmingham, Centre for Patient Reported Outcomes Research and Institute of Applied Health Research

**Primary Subject Heading**:	Patient-centred medicine
Secondary Subject Heading:	Health services research
Keywords:	Quality in health care < HEALTH SERVICES ADMINISTRATION & MANAGEMENT, Organisation of health services < HEALTH SERVICES ADMINISTRATION & MANAGEMENT, Protocols & guidelines < HEALTH SERVICES ADMINISTRATION & MANAGEMENT

SCHOLARONE™
Manuscripts

I, the Submitting Author has the right to grant and does grant on behalf of all authors of the Work (as defined in the below author licence), an exclusive licence and/or a non-exclusive licence for contributions from authors who are: i) UK Crown employees; ii) where BMJ has agreed a CC-BY licence shall apply, and/or iii) in accordance with the terms applicable for US Federal Government officers or employees acting as part of their official duties; on a worldwide, perpetual, irrevocable, royalty-free basis to BMJ Publishing Group Ltd ("BMJ") its licensees and where the relevant Journal is co-owned by BMJ to the co-owners of the Journal, to publish the Work in this journal and any other BMJ products and to exploit all rights, as set out in our licence.

The Submitting Author accepts and understands that any supply made under these terms is made by BMJ to the Submitting Author unless you are acting as an employee on behalf of your employer or a postgraduate student of an affiliated institution which is paying any applicable article publishing charge ("APC") for Open Access articles. Where the Submitting Author wishes to make the Work available on an Open Access basis (and intends to pay the relevant APC), the terms of reuse of such Open Access shall be governed by a Creative Commons licence – details of these licences and which Creative Commons licence will apply to this Work are set out in our licence referred to above.

Other than as permitted in any relevant BMJ Author's Self Archiving Policies, I confirm this Work has not been accepted for publication elsewhere, is not being considered for publication elsewhere and does not duplicate material already published. I confirm all authors consent to publication of this Work and authorise the granting of this licence.

For peer review only

Protocol for a scoping review exploring the use of patient reported outcomes in adult social care

Sarah E. Hughes[1,2]
Olalekan L. Aiyegbusi[1,2,3]
Dan Lasserson[2,4,5]
Philip Collis[1]
Samantha Cruz-Rivera[1,7]
Christel McMullan[1]
Grace Turner[1,3]
Jon Glasby[6]
Melanie Calvert[1,2,3,7]

[1]Centre for Patient Reported Outcome Research, Institute of Applied Health Research, University of Birmingham B15 2TT UK

[2]National Institute of Health Research (NIHR) Applied Research Centre (ARC) West Midlands, Institute of Applied Health Research, University of Birmingham B15 2TT UK

[3]NIHR Birmingham Biomedical Research Centre, NIHR Surgical Reconstruction and Microbiology Research Centre, University of Birmingham B15 2TT UK

[4]Warwick Medical School, University of Warwick, Coventry, CV4 7AL.

[5]Department of Acute Medicine, City Hospital, Sandwell and West Birmingham NHS Trust, Birmingham B18 7QH UK

[6]Department of Social Work and Social Care, University of Birmingham B15 2TT

[7]Birmingham Health Partners Centre for Regulatory Science and Innovation, University of Birmingham B15 2TT UK

Address for correspondence:
Sarah Hughes
Centre for Patient Reported Outcome Research (CPROR)
Institute of Applied Health Research, University of Birmingham
Edgbaston, Birmingham, UK
Tel: +44 121 414 3353
E-mail: s.e.hughes@bham.ac.uk

For peer review only

ABSTRACT

Introduction - Patient reported outcomes (PROs) are measures of a person's own views of their health, functioning, and quality of life. They are typically assessed using validated, self-completed questionnaires known as patient reported outcome measures (PROMs). PROMs are used in healthcare settings to support care planning, clinical decision-making, patient-practitioner communication, and quality improvement. PROMs have a potential role in the delivery of social care where people often have multiple and complex long-term health conditions. However, the use of PROMs in this context is currently unclear. The objective of this scoping review is to explore the evidence relating to the use of PROMs in adult social care.

Methods and analyses - The electronic databases Medline (Ovid), PsychInfo (Ovid), ASSIA (ProQuest), Social Care Online (SCIE), Web of Science, and EMBASE (Ovid) were searched on 29th September 2020 to identify eligible studies and other publically available documents published since 2010. A grey literature search and hand searching of citations and reference lists of the included studies will also be undertaken. No restrictions on study design or language of publication will be applied. Screening and data extraction will be completed independently by two reviewers. Quality appraisal of the included documents will use the CASP and AACODS checklists. A customised data charting table will be used for data extraction, with analysis of qualitative data using the Framework Method. The review findings will be presented as tables and in a narrative summary.

Ethics and dissemination - Ethical review is not required as scoping reviews are a form of secondary data analysis that synthesise data from publically available sources. Review findings will be shared with service users and other relevant stakeholders and disseminated through a peer-reviewed publication and conference presentations. This protocol is registered on the Open Science Framework (www.osf.io).

For peer review only - http://bmjopen.bmj.com/site/about/guidelines.xhtml

STRENGTHS AND LIMITATIONS

- This scoping review will follow the PRISMA guidelines for the conduct of scoping reviews, ensuring transparency and rigour.
- Service user and community involvement in the design and conduct of the review will ensure the findings are supported by stakeholder experiences, further helping to identify evidence gaps and research priorities.
- An appraisal of the methodological quality of studies is not a requirement of a scoping review; however, a quality appraisal of the included research articles will be conducted to gather insights into the types, sources, and quality of the evidence around the use of PROs in adult social care.

For peer review only

For peer review only - http://bmjopen.bmj.com/site/about/guidelines.xhtml

INTRODUCTION

People with care and support needs often live with multiple and complex, long term health conditions.[1] They are likely to experience greater levels of disability and have complex care and support needs.[2 3] An understanding of a person's own views about their health can help ensure care and support is provided in a way that is holistic, person-centred, and responsive. Such insights are particularly relevant in the UK where a fragmented health and social care system makes the coordination of care challenging.[4]

Individuals' perspectives about their health are most useful to care providers when these first-hand accounts are both relevant and reliably measured.[5] A patient reported outcome (PRO) is "a measurement of any aspect of a patient's health that comes directly from the patient without the interpretation of the patient's responses by anyone else".[6] PROs include symptom severity of the impact of a disease or its treatment on a patient's health-related quality of life (including functioning and social and emotional well-being).[7] PROs are typically measured using self-report questionnaires known as patient reported outcome measures (PROMs).

PROs are implemented widely across different levels of health care.[8] At an individual level, PROs may be used to inform decision-making around treatment, monitor disease symptoms and treatment adherence, and facilitate communication between the patient and their clinical team. At a systems level, PROs may be used support benchmarking including service evaluation and commissioning of services, inform policy-making, and support quality improvement initiatives. PROs are used as endpoints within clinical trials, support regulatory approval of new medicinal products and devices, and inform clinical guidelines and health policy.[5 9 10] PROs are valued due their capability to ensure the patient's unique perspective is represented in the measurement of health and when capturing the effectiveness of healthcare interventions.

Despite their established use in healthcare, the role of PROs and PROMs in social care is less clear. Published reviews have tended to study PROMs within a specific context, health condition, or population. For example, reviews have explored the use of PROMs in primary care and investigated stakeholders' perspectives regarding the use of PROMs with frail, older adults living at home.[11 12] Reviews have also been conducted that focus on the use of PROMs in end-of-life care and in the care of people with dementia and other long-term conditions.[13-16] To date, a review focussing on the use of PROMs in the broader context of adult social care has not been identified.

The Care Act (2014) places a duty upon Local Authorities to ensure that the wellbeing of individuals is placed at the heart of care and support.[17] Commensurately, in social care,

For peer review only - http://bmjopen.bmj.com/site/about/guidelines.xhtml

PROMs have focussed on measuring the wider determinants of well-being (e.g., personal dignity, control by an individual, participation in work or training, and mental, physical and emotional health) and social-care related quality of life.[18] However, it has been suggested that health, as a component of well-being, may be more appropriately measured with health-related PROMs as a complement existing social care measures.[19 20] A better understanding of how health and social care-related PROMs are utilised in the delivery of care and support, by whom, and the barriers and facilitators to their utilisation, may be considered increasingly relevant as decision-making around care and support becomes more person-focussed and data driven.[21] A comprehensive understanding of PROs may be particularly pertinent as health, care and support services move towards greater integration.[22] Clarification on PROM use is especially timely in light of the Covid-19 pandemic, which has resulted in the increased use of telemedicine, virtual consultations, and remote assessment and monitoring.[23 24] As such, PROMs have the potential to help people with care and support needs express their views about their health (including symptoms, functioning, and quality of life) and well-being.[25]

Consistent with the remit of scoping reviews as a form of knowledge synthesis, the aim of this scoping review is to explore the existing evidence to understand the current state of knowledge on the use of PROs in adult social care. To achieve this aim, the specific review objectives are: 1) to identify PROMs used by people to meet their own direct care and support needs; 2) to characterise how PROMs have been used in this context; and to chart the evidence relating to 3) their effectiveness; 4) barriers and facilitators to their implementation; and 5) stakeholder views on their use. A sub-analysis to explore the use of PROMs in the delivery of integrated health and social care will also be undertaken.[26]

A shared language for PROs

The term "patient-reported outcome" is a reflection of PROs' origins in medicine and healthcare. However, in the context of social care people with care and support needs rarely identify as patients, preferring to identify as service users or citizens.[27] In this review on the use of PROs within adult social care, the term "person-reported outcome" and "person-reported outcome measure" will be used.

METHODS AND ANALYSIS

Protocol design, registration, and reporting

Scoping reviews are conducted to understand the existing research on a given topic and often serve as a precursor to systematic reviews.[28] Specifically, scoping reviews seek to identify

For peer review only - http://bmjopen.bmj.com/site/about/guidelines.xhtml

and map the available evidence and, in so doing, identify knowledge gaps and directions for future research.[29] Frequently, scoping reviews are complemented by stakeholder consultation exercises to ensure a holistic understanding of the topic that is informed by both the published evidence and lived experience.[30] This scoping review will use the methodology proposed by the Joanna Briggs Institute, supported by the original scoping framework proposed by Arksey and O'Malley.[30 31] The Preferred Reporting Items for Systematic Reviews and Meta-analyses guidance for protocol development (PRISMA-P) and scoping reviews (PRISMA-ScR) and JBI reporting checklists were consulted when preparing this protocol.[31-33] This review protocol is registered online with the Open Science Framework (www.osf.io).

Eligibility criteria

Participants

The review will consider sources of evidence that report on adults (≥ 18 years of age) who are direct users of social care services or receive integrated health and social care. No limitations with regard to an individual's health condition(s) will be applied.

Concept

Sources of evidence that provide information on any PROM used in integrated or social care will be considered for inclusion in the review. Self-report instruments that are proxy-reported and carer-reported measures will be excluded.

Context

Sources of evidence reporting on a social care or integrated care intervention will be considered. An intervention may be defined as an "action where someone gets involved to improve a situation or prevent it getting worse" that is undertaken with, or on behalf of, a service user to promote independence, provide support, prevent harm and enable them to live their lives in accordance with their own wishes and beliefs.[34 35] Integrated care refers to coordinated health and social care that is planned and organised around the needs and preferences of the individual, their carers and family.[22 34] The review will exclude studies and other evidence sources set in primary and secondary care contexts.

Types of sources

This scoping review will consider all sources of published evidence on the use of PROs and PROMs in adult social care. Sources of evidence will include primary research studies, websites, guidelines, government reports, and policy-documents. Expert opinions, editorials, and studies reporting secondary data analysis (i.e., systematic reviews) will also be included.

For peer review only - http://bmjopen.bmj.com/site/about/guidelines.xhtml

Information sources

The searches will be conducted in three stages: 1) electronic database searching; 2) grey literature searching; and 3) hand searching the reference lists and citations of included sources to identify further studies for inclusion. Electronic database selection will be based on recommendations for optimal database searching for sensitivity.[36]

The electronic databases Medline (Ovid), Embase (Ovid), PsychInfo (Ovid), HMIC (Ovid), Social Care Online (SCIE), ASSIA (Pro Quest) and Web of Science (Pro Quest) were searched on 29th September 2020 for the period 2010 to present date, with no language limitation. Sources published in languages other than English will be translated. Grey literature databases, Google, and websites of government and third sector organisations will be searched to locate relevant sources. Hand searching of all of the included sources' reference lists will also be undertaken. Grey literature will be defined as "work that is produced on all levels of government, academia, business, and industry in print and electronic formats but which is not controlled by commercial publishers.[37] A ten-year date limit on searches was considered appropriate for locating relevant sources, reflecting the increased use of PROMs over the last decade, the introduction of the UK National PROMs programme, publication of the FDA's guidance on PROMs, and the UK government's NHS white paper outlining government's vision for greater integration of health and social care.[6 38 39]

Search strategy

The Medline search strategy will be developed and then adapted for use with other databases. The search strategy will combine MeSH terms (and relevant synonyms) and the Boolean operators "AND" and "OR" for search strings. Four concept clusters will be included: 1) the concept of interest (i.e., PROs and PROMs); 2) the population of adults who use social care or integrated care services; 3) contextual factors including type of integrated care/social care intervention and 4) PROM implementation (e.g., barriers and facilitators). Terms were developed by the review team with support from an information science specialist (SB). Service user partner and co-investigator (PC) will be consulted to identify specific search terms related to integrated care and social care. The grey literature search will take place after searches of the electronic databases have been completed to enable to study team to immerse themselves in the literature and come to better understand the evidence base. Hand searching of citations and reference lists of included documents will be undertaken at each stage of the search process. The Medline search strategy is presented in Supplementary file 1.

For peer review only - http://bmjopen.bmj.com/site/about/guidelines.xhtml

Selection of sources of evidence

A team of reviewers (SH, GT, CM, SCR) will conduct title/abstract and full-text screening of all records against pre-specified eligibility criteria to ascertain suitability for inclusion in the review. All records will be screened independently by a minimum of two reviewers. Disagreements between reviewers will be resolved through discussion. If necessary, a third reviewer will be involved if consensus cannot be reached. The eligibility criteria will be pre-tested on a sample of abstracts to ensure that evidence sources discussing the use of PROs and PROMs in integrated/social care are captured. Records identified from electronic databases, the grey literature, and hand searching will be imported to Endnote 9.3.3 (www.endnote.com) reference management software and duplicate records will be removed. All screening will be conducted within Endnote. Any retrieved documents deemed to meet the inclusion criteria will go forward for full-text review. For articles excluded during full text screening, a reason for exclusion will be documented. Disagreements on article inclusion will be resolved through discussion by members of the review team until consensus is achieved. The study selection process will be documented in a PRISMA flow diagram.

Critical appraisal of individual sources of evidence

Assessment of the methodological quality of included studies is not a requirement of scoping reviews.[31] The decision to undertake a quality appraisal will be governed by the number and type of sources identified by the searches. If feasible, a formal quality assessment will be undertaken using the Critical Appraisal Skills Programme (CASP) checklists and the AACODS checklist for quality appraisal of the grey literature.[40 41] Appraisals will be undertaken to characterise the overall quality of the evidence base and no study will be excluded on the basis of its methodological quality. Two reviewers will independently appraise studies for quality. Findings will be collated by study type and presented in tabular format along with a textual description of findings. To ensure transparency of reporting, interrater agreement and reliability will be determined by calculating an intra-class coefficient (ICC) statistic, Cohen's kappa (κ), and percentage agreement.[42 43]

Data charting process

Data extraction, termed data charting in scoping reviews, aims to provide a "logical and descriptive summary of results that aligns with the review questions".[31] A standardised template will be developed in Excel and piloted prior to commencing data charting. Two

For peer review only - http://bmjopen.bmj.com/site/about/guidelines.xhtml

reviewers will extract key characteristics of the data source and information relevant to answering the review questions independently. Discrepancies will be resolved through discussion. As a scoping review is an iterative process, the data extraction form will be refined and updated throughout the course of the review and in response to the emergent findings.

Data items

The data items to be extracted for each data source comprise: 1) the publication title; 2) date of publication; 3) authors; 4) country (location of study); 5) study aims and objectives, 6) study design; 7) study setting; 8) whether the intervention is an integrated care intervention); 9) study population; 10) study data collection method; 11) data analysis method. Information on community and user involvement, study outcome(s), process and system-level outcomes in response to the PROM intervention, and barriers/enablers to implementation will also be extracted. PROM-specific information will be charted separately and include a list of studies using each PROM and, if available, a description of the PROMs' characteristics, including target population, construct domains, number of items/subscales, mode of administration, recall period, response options, range of scores, scoring, language of the original publication and available translations.

Data summary

A qualitative content analysis in scoping reviews is generally descriptive in nature.[31] Following data charting, findings from the included sources will be collated with analysis of common themes relating to the aims of the review. Given the anticipated diversity of the included data sources, the framework method, using NVivo (Version 1.3) qualitative data analysis software and Microsoft Excel spreadsheets, and data charting will be used to capture key themes.[44 45] The framework method sits within in the family of methods described as thematic analysis or content analysis. Its hallmark is the systematic construction of a matrix that facilitates comparison across and within cases (i.e., data sources) to produce a thematic interpretation of the data. The framework method follows a structured format whereby the reviewers will first become familiar with the data source. The data will be reviewed line-by-line and coded by applying a paraphrase or label. Coding will include a number of *a priori* deductive codes selected for their relevance to the aims of this scoping review to chart the evidence of PROM use in adult social care. The reviewer will also deploy inductive coding by remaining open to the coding of new and unexpected concepts that may be emergent in the data. The first few data sources will be coded independently by two reviewers. Through

For peer review only - http://bmjopen.bmj.com/site/about/guidelines.xhtml

consensus discussions, the reviewers will agree on a set of codes to be applied to all subsequent sources. Codes will be grouped into categories to create an analytical framework that will be used for indexing the remaining data sources. Lastly, the coded data will be charted into a matrix using a spreadsheet. The characteristics of and differences between the data will be identified and a description of relationships between categories will be developed. Results will be presented using tables and diagrams accompanied by a narrative summary.[31]

Piloting/Calibration exercise

All phases of the scoping review will be pretested. The JBI recommended procedure for pilot testing will be used.[31] For screening, the titles and abstracts of 25 randomly selected articles will be screened by the review team, applying the *a priori* review eligibility criteria. Screening will commence once inter-rater agreement of 75% or greater is achieved. Inter-rater agreement that is less than 75% will prompt the study team to review and refine the inclusion criteria. This process of pilot testing will continue iteratively until the 75% agreement threshold is met.

The data charting template will be piloted by two reviewers to ensure the capture of data relevant to the aims and objective of the review. As the review progresses, the reviewers may wish to extract additional information of relevance to the review objectives; therefore, further revisions to the data chart template may be made iteratively as the review progresses.[31]

Changes to the protocol

The exploratory nature and breadth of a scoping review mean that changes to the protocol may be required as new information becomes available and as the review progresses. Any protocol deviations or refinements, together with a rationale for these changes, will be documented clearly in the review report.

Service user and public involvement

This scoping review was conceived and developed with community and user involvement. As co-investigator and a person with lived experience, PC contributed to the development of the search strategies, the study protocol, consensus discussions, and will review all study-related documents. A lay summary to facilitate dissemination of the review findings will be coproduced. In addition, this work will be discussed with the NIHR ARC West Midlands Long Term Conditions Theme 1 Patient and Public Involvement and Engagement

For peer review only - http://bmjopen.bmj.com/site/about/guidelines.xhtml

(PPIE) Stakeholder Advisory Group. Meetings will be held on a quarterly basis throughout the review process. The Guidance for Reporting Involvement of Patients and the Public-2 short form (GRIPP-2SF) checklist will be used to document service user and public involvement.[46]

Ethics and dissemination

Ethical approval is not required as this review is a retrospective review of publicly available evidence sources. The review findings will be disseminated via publication in a peer-reviewed journal, symposia, and conference presentations. A dissemination strategy to ensure the review findings reach relevant stakeholders will be coproduced in partnership with members of the review team who are experts by experience.

DISCUSSION

PROs can provide key data that enable health and social care practitioners to understand better a person's views about their health and wellbeing, allowing them to provide effective, efficient, and compassionate care. This scoping review will be the first to provide an overview of the evidence around the use of PROs with adults who use social care services. Given the disparate nature of the evidence, the use of a scoping review methodology is appropriate as it enables the review to evolve in an iterative manner that is responsive to the evidence.

REFERENCES

1. Goodwin N, Curry N, Naylor C, et al. Managing People with Long Term Conditions. London: The King's Fund 2010.
2. Vogeli C, Shields AE, Lee TA, et al. Multiple Chronic Conditions: Prevalence, Health Consequences, and Implications for Quality, Care Management, and Costs. *J Gen Int Med* 2007;22(3):391-95. doi: 10.1007/s11606-007-0322-1
3. Barnett K, Mercer SW, Norbury M, et al. Epidemiology of multimorbidity and implications for health care, research, and medical education: a cross-sectional study. *Lancet* 2012;380(9836):37-43. doi: 10.1016/S0140-6736(12)60240-2
4. Glasby J. The holy grail of health and social care integration. *BMJ* 2017;356:j801. doi: 10.1136/bmj.j801
5. FDA. Principles for Selecting, Developing, Modifying, and Adapting PatientReported Outcome Instruments for Use in Medical Device Evaluation: Draft Guidance for Industry and Food and Drug Administration Staff, and Other Stakeholders. 2020. (accessed 31 August 2020).
6. FDA. Guidance for industry: Patient-reported outcome measures: Use in medicinal product development to support labelling claims, 2009.
7. Wilson IB. Linking clinical variables with health-related quality of life. A conceptual model of patient outcomes. *JAMA* 1995;273(1):59-65. doi: 10.1001/jama.273.1.59

For peer review only - http://bmjopen.bmj.com/site/about/guidelines.xhtml

8. Calvert M, Kyte D, Price G, et al. Maximising the impact of patient reported outcome assessment for patients and society. *BMJ* 2019;364:k5267. doi: 10.1136/bmj.k5267
9. Kyte D, Duffy H, Fletcher B, et al. Systematic evaluation of the patient-reported outcome (PRO) content of clinical trial protocols. *PloS One* 2014;9(10):e110229-e29. doi: 10.1371/journal.pone.0110229
10 FDA. Principles for Selecting, Developing, Modifying, and Adapting Patient-reported Outcome Instruments for Use in Medical Device Evaluation: Draft Guidance for Industry and Food and Drug Administration Staff, and Other Stakeholders: US Food and Drug Administration 2020.
11. Murphy M, Hollinghurst S, Salisbury C. Identification, description and appraisal of generic PROMs for primary care: a systematic review. *BMC Fam Pract* 2018;19(1):41. doi: 10.1186/s12875-018-0722-9
12. Schick-Makaroff K, Karimi-Dehkordi M, Cuthbertson L, et al. Using Patient- and Family-Reported Outcome and Experience Measures Across Transitions of Care for Frail Older Adults Living at Home: A Meta-Narrative Synthesis. *Gerontologist* 2020 doi: 10.1093/geront/gnz162
13. Kearns T, Cornally N, Molloy W. Patient reported outcome measures of quality of end-of-life care: A systematic review. *Maturitas* 2017;96:16-25. doi: 10.1016/j.maturitas.2016.11.004
14. Johnston BM, Flemming K, Narayanasamy MJ, et al. Patient reported outcome measures for measuring dignity in palliative and end of life care: a scoping review. *BMC Health Serv Res* 2017;17
15. Ayton DR, Gardam ML, Pritchard EK, et al. Patient-Reported Outcome Measures to Inform Care of People With Dementia—A Systematic Scoping Review. *Gerontologist* 2020 doi: 10.1093/geront/gnz179
16. Forestier B, Anthoine E, Reguiai Z, et al. A systematic review of dimensions evaluating patient experience in chronic illness. *Health Qual Life Outcomes* 2019;17(1):19-19. doi: 10.1186/s12955-019-1084-2
17. UK Government. The Health and Social Care Act 2008 (Regulated Activities) Regulations 2014, 2014.
18. Malley J, Netten A. Measuring outcomes of social care. *Research, Policy, and Planning* 2009;27(2):85-96.
19. Hackert MQN, van Exel J, Brouwer WBF. Does the ICECAP-O cover the physical, mental and social functioning of older people in the UK? *QualLife Res* 2019;28(3):761-70. doi: 10.1007/s11136-018-2042-x
20. Makai P, Brouwer WB, Koopmanschap MA, et al. Quality of life instruments for economic evaluations in health and social care for older people: a systematic review. *Soc Sci Med* 2014;102:83-93. doi: 10.1016/j.socscimed.2013.11.050
21. Fund TKs. How to develop evidence-based solutions to improve patient journeys: The King's Fund, 2020.
22. National Collaboration for Integrated Care and Support. Integrated Care and Support2013.
23. Greenhalgh T, Koh GCH, Car J. Covid-19: a remote assessment in primary care. *BMJ* 2020;368:m1182. doi: 10.1136/bmj.m1182
24. Aiyegbusi OL, Calvert MJ. Patient-reported outcomes: central to the management of COVID-19. *Lancet* 2020;396(10250):531. doi: 10.1016/s0140-6736(20)31724-4
25. Taylor PC. Adopting PROs in virtual and outpatient management of RA. *Nat Rev Rheumatol* 2020;16(9):477-78. doi: 10.1038/s41584-020-0449-6

For peer review only - http://bmjopen.bmj.com/site/about/guidelines.xhtml

26. Humphries R. Integrated health and social care in England – Progress and prospects. *Health Policy* 2015;119(7):856-59. doi: https://doi.org/10.1016/j.healthpol.2015.04.010
27. Glasby J. Understanding Health and Social Care. Revised, 3rd ed. Bristol University Press 2017.
28. Peterson J, Pearce PF, Ferguson LA, et al. Understanding scoping reviews: Definition, purpose, and process. *J Am Assoc Nurse Pract* 2017;29(1)
29. Munn Z, Peters MDJ, Stern C, et al. Systematic review or scoping review? Guidance for authors when choosing between a systematic or scoping review approach. *BMC Med Res Methodol* 2018;18(1):143. doi: 10.1186/s12874-018-0611-x
30. Arksey H, O'Malley L. Scoping studies: towards a methodological framework. *International Journal of Social Research Methodology* 2005;8(1):19-32. doi: 10.1080/1364557032000119616
31. Peters M, Godfrey C, McInerney P, et al. Chapter 11: Scoping Reviews (2020 Version). In: Aromataris E, Munn Z, eds. JBI Manual for Evidence Synthesis: JBI 2020.
32. Moher D, Shamseer L, Clarke M, et al. Preferred reporting items for systematic review and meta-analysis protocols (PRISMA-P) 2015 statement. *Syst Rev* 2015;4(1):1. doi: 10.1186/2046-4053-4-1
33. Tricco AC, Lillie E, Zarin W, et al. PRISMA Extension for Scoping Reviews (PRISMA-ScR): Checklist and Explanation. *Ann Intern Med* 2018;169(7):467-73. doi: 10.7326/m18-0850
34. Think Local Act Personal. TLAP Care and Support Jargon Buster. https://www.thinklocalactpersonal.org.uk/Browse/Informationandadvice/CareandSupportJargonBuster/ (accessed 27 Jan 2021)
35. Harris J, White V. Oxford Dictionary of Social Work and Social Care. 2nd Ed. Oxford: Ocford University Press 2018.
36. Bramer WM, Rethlefsen ML, Kleijnen J, et al. Optimal database combinations for literature searches in systematic reviews: a prospective exploratory study. *Syst Rev* 2017;6(1):245. doi: 10.1186/s13643-017-0644-y [published Online First: 2017/12/07]
37. Godin K, Stapleton J, Kirkpatrick SI, et al. Applying systematic review search methods to the grey literature: a case study examining guidelines for school-based breakfast programs in Canada. *Syst Rev* 2015;4(1):138. doi: 10.1186/s13643-015-0125-0
38. Devlin NJ, Appleby J. Getting the Most Out of PROMs. London: The King's Fund, 2010.
39. Equity and Excellence: Liberating the NHS. In: Health Do, ed. London: HMSO, 2010.
40. CASP Checklists 2020 [Available from: https://casp-uk.net/casp-tools-checklists/ (accessed 28 August 2020)
41. Tyndall J. ACCODS checklist for appraising grey literature. 2010. https://dspace.flinders.edu.au/jspui/bitstream/2328/3326/4/AACODS_Checklist.pdf (accessed 20 September 2020)
42. Shrout PE, Fleiss JL. Intraclass correlations: uses in assessing rater reliability. *Psychol Bull* 1979;86(2):420-8. doi: 10.1037//0033-2909.86.2.420
43. Cohen J. A coefficient of agreement for nominal scales. *Educ Psychol Meas* 1960;20:37-46. doi: 10.1177/001316446002000104
44. Gale NK, Heath G, Cameron E, et al. Using the framework method for the analysis of qualitative data in multi-disciplinary health research. *BMC Med Res Methodol* 2013;13:117. doi: 10.1186/1471-2288-13-117
45. Ritchie J, Spence L. Qualitative data analysis for applied policy research. In: Bryman A, Burgess RG, eds. Analyzing Qualitative Data. New York: Routledge 1994

For peer review only - http://bmjopen.bmj.com/site/about/guidelines.xhtml

46. Staniszewska S, Brett J, Simera I, et al. GRIPP2 reporting checklists: tools to improve reporting of patient and public involvement in research. *Res Involv Engagem* 2017;3(1):13. doi: 10.1186/s40900-017-0062-2

Acknowledgments

The authors wish to thank Sue Bayliss, Information Specialist, University of Birmingham for her assistance in developing the search strategy.

Contributors

SH, MC, OLA, DL, JG conceptualised the study and contributed to the study design. SH and PC developed the search strategy. GT, SCR, CM contributed to methods design. SH drafted and edited the manuscript with MC, OLA, DL, JG, PC, GT, SCR, CM providing critical revisions to the manuscript and supplementary material. The final version was read and approved by all authors (SH, MC, OLA, DL, JG, PC, GT, SCR, CM).

Funding

This research is funded by the National Institute for Health Research (NIHR) Applied Research Collaboration (ARC) West Midlands. The views expressed are those of the author(s) and not necessarily those of the NIHR or the Department of Health and Social Care.

Competing interests

SH is supported by the National Institute of Health Research (NIHR) Applied Research Centre (ARC), West Midlands. SH is company director of Narra Consulting Limited and undertakes consultancy work for Cochlear Ltd. MC is a National Institute for Health Research (NIHR) Senior Investigator and receives funding from the National Institute for Health Research (NIHR) Birmingham Biomedical Research Centre, the NIHR Surgical Reconstruction and Microbiology Research Centre and NIHR ARC West Midlands at the at the University of Birmingham and University Hospitals Birmingham NHS Foundation Trust, Health Data Research UK, Innovate UK (part of UK Research and Innovation), Macmillan Cancer Support, UCB Pharma MC has received personal fees from Astellas, Takeda, Merck, Daiichi Sankyo, Glaukos, GSK and the Patient-Centered Outcomes Research Institute (PCORI) outside the submitted work. OLA is supported by the National Institute of Health Research (NIHR) Birmingham Biomedical Research Centre (BRC), Birmingham, West Midlands. OLA also receives funding from the Health Foundation and has received personal fees from Gilead Sciences. DL is supported by the NIHR ARC West Midlands and the NIHR Community Healthcare MedTech and IVD Cooperative, hosted by Oxford Health NHS Foundation Trust. JG is a non-executive director of University Hospitals Birmingham, adjunct professor at Curtin University, member of the leadership team for an ESRC large grant on sustainable adult social care, and member of the expert advisory group for West Midlands ARC. PC, GT, SCR, and CM have no conflicts of interest to declare.

Provenance and peer review

Not commissioned; externally peer reviewed.

Patient consent for publication

Not required

For peer review only - http://bmjopen.bmj.com/site/about/guidelines.xhtml

Data sharing statement

No additional data are available.

Open access

This is an Open Access article distributed in accordance with the Creative Commons Attribution Non Commercial (CC-BY-NC 4.0) license, which permits others to distribute, remix, adapt, build upon this work non-commercially, and license their derivative works on different terms, provided the original work is properly cited and the use is non-commercial. See: http://creativecommons.org/licenses/by-nc/4.0/.

For peer review only

Supplemental File 1 – Search strategy

Note: Lines 48-72 are based on the modified Oxford PROM filter and the search strategy developed by Aiyegbusi et al.[1]

	**Setting**
1	respite care/
2	respite care.ti,ab.
3	hospice/ or home care/ or palliative care/ or terminally ill patients/
4	exp client transfer/ or exp facility discharge/ or exp hospital discharge/ or exp patient selection/ or exp professional referral/ or exp psychiatric hospital discharge/ or exp treatment refusal/ or exp treatment termination/
5	patient transfer.ti,ab.
6	hospice.ti,ab.
7	residential home.ti,ab.
8	care home*.ti,ab.
9	aged care home*.ti,ab.
10	nursing homes/
11	retirement home*.ti,ab.
12	adult day care centre*.ti,ab.
13	homes for the aged.ti,ab.
14	self-care skills/ or aging in place/ or independent living programs/ or rehabilitation/
15	independent living.ti,ab.
16	personalised budget*.ti,ab.
17	reablement.ti,ab.
18	home nursing.ti,ab.
	**Population**
19	aged.ti,ab.
20	geriatric patients/
21	old* person.ti,ab.
22	(elderly or geriatric).ti,ab.
23	exp Disabilities/ or exp Intellectual Development Disorder/ or exp Developmental Disabilities/
24	disabled person*.ti,ab.
25	disabled people.ti,ab.
26	(disabled adj3 person*).ti,ab.
27	vulnerable population*.ti,ab.
28	vulnerable person*.ti,ab.
29	intellectual development disorder/
30	exp Autism Spectrum Disorders/
31	autism spectrum disorder.ti,ab.
32	intellectual disabilit*.ti,ab.
33	learning disabilit*.ti,ab.
34	exp Chronic Illness/ or exp Long Term Care/

For peer review only

35	long term condition*.ti,ab.
36	chronic illness.ti,ab.
37	exp substance-related disorders/
38	mental* ill*.ti,ab.
39	drug user.ti,ab.
40	homeless.ti,ab.
41	clients/
42	service user*.ti,ab.
43	adult.ti,ab.
	**Type of Care**
44	exp Social Services/
45	social care.ti,ab.
46	integrated services/
47	integrated care.ti,ab.
	**PROs**
48	(PRO integration or Clinical PRO application* or telePRO or automated PRO algorithm* or screening purpose* or PRO questionnaire* or Patient-reported outcome questionnaire* or PROM or Patient-reported outcome measure* or Patient reported outcome measure* or Patient-reported symptom* or Patient-centred care or Patient self-report* or Self-report health or Self-rated health or Self-reported measure* of health or Health outcome* or Health communication* or Hospital performance evaluation* or Automated telephone survey system* or paper-based survey* or web-based survey* or web-based PRO platform* or web-based system* or PRO collection* or PRO measure* or PRO intervention* or PRO assessment intervention* or PRO data or PRO assessment* or Routine PRO assessment* or Routine PRO collection or Symptom assessment* or Symptom monitoring or Symptom data or Functional status or Electronic PRO assessment* or Electronic PRO system* or ePRO or ePRO* or ePRO system* or PRO system* or Generic PRO system* or PRO-based clinical alert system*).ti,ab.
49	exp Patient Reported Outcome Measures/
50	((((patient adj reported adj outcome adj measure*) or patient) adj reported adj outcome*) or capability or capabilities).ti,ab.
51	((disability or function or functional or functions or subjective or utility or utilities or wellbeing or well being) adj2 (index or indices or instrument or instruments or measure or measures or questionnaire* or profile or profiles or scale or scales or score or scores or status or survey or surveys)).ti,ab.
52	((patient or self or carer or proxy) adj (appraisal* or appraised or report or reported or reporting or rated or rating or based or assessed or assessment*)).ti,ab.
53	self-report/
54	health status/
55	(health index* or health indices or health status or health profile*).ti,ab.
56	exp "Quality of Life"/
57	quality of life.ti,ab.
58	(HR-PRO or HRPRO or HRQL or HRQoL or QL or QoL).ti,ab.

For peer review only

59	(Patient Centred Care or person centred care or person centred coordinated care or p3c or person centred outcome*).ti,ab. or person reported outcome*.mp. or individualised patient reported outcome measur*.ti,ab. or iprom.ti,ab.
60	patient centered care.ti,ab.
61	person centered care.ti,ab.
62	(Patient Centered Care or person centered care or person centered coordinated care or p3c or person centred outcome*).ti,ab. or person reported outcome*.mp. or individualised patient reported outcome measur*.ti,ab. or iprom.ti,ab.
	**Implementation**
63	((implementation or implementation science).ti,ab. or implementation.mp.) adj3 science.ti,ab.
64	"barriers and facilitators".ti,ab.
65	1 or 2 or 3 or 4 or 5 or 6 or 7 or 8 or 9 or 10 or 11 or 12 or 13 or 14 or 15 or 16 or 17 or 18
66	19 or 20 or 21 or 22 or 23 or 24 or 25 or 26 or 27 or 28 or 29 or 30 or 31 or 32 or 34 or 35 or 36 or 41 or 42 or 43
67	44 or 45 or 46 or 47
68	48 or 49 or 50 or 51 or 52 or 53 or 54 or 55 or 56 or 57 or 58 or 59 or 60 or 61 or 62
69	65 and 66 and 67 and 68
70	limit 69 to yr="2010 -Current"

References:

1. Aiyegbusi OL, Kyte D, Cockwell P, et al. Measurement properties of patient-reported outcome measures (PROMs) used in adult patients with chronic kidney disease: a systematic review protocol. *BMJ Open* 2016;6(10):e012014. doi: 10.1136/bmjopen-2016-012014

For peer review only

PRISMA-P 2015 Checklist

This checklist has been adapted for use with protocol submissions to *Systematic Reviews* from Table 3 in Moher D et al: Preferred reporting items for systematic review and meta-analysis protocols (PRISMA-P) 2015 statement. *Systematic Reviews* 2015 **4**:1

Section/topic	#	Checklist item	Information reported: Yes	Information reported: No	Line number(s)
**ADMINISTRATIVE INFORMATION**					
**Title**					
Identification	1a	Identify the report as a protocol of a scoping review	X	☐	2 (Line 45)
Update	1b	If the protocol is for an update of a previous systematic review, identify as such	☐	☐	N/A
**Registration**	2	If registered, provide the name of the registry (e.g., PROSPERO) and registration number in the Abstract	X	☐	2 (Lines 59-60)
**Authors**					
Contact	3a	Provide name, institutional affiliation, and e-mail address of all protocol authors; provide physical mailing address of corresponding author	X	☐	1 (Lines 14-35)
Contributions	3b	Describe contributions of protocol authors and identify the guarantor of the review	X	☐	14
**Amendments**	4	If the protocol represents an amendment of a previously completed or published protocol, identify as such and list changes; otherwise, state plan for documenting important protocol amendments	☐	☐	N/A
**Support**					
Sources	5a	Indicate sources of financial or other support for the review	X	☐	14 Lines 456-458)
Sponsor	5b	Provide name for the review funder and/or sponsor	X	☐	14 (Lines 456-458)
Role of sponsor/funder	5c	Describe roles of funder(s), sponsor(s), and/or institution(s), if any, in developing the protocol	X	☐	14 (Lines 456-458)
**INTRODUCTION**					
**Rationale**	6	Describe the rationale for the review in the context of what is already known	X	☐	4-5 (Lines 85-126)
**Objectives**	7	Provide an explicit statement of the question(s) the review will address with reference to participants, interventions, comparators, and outcomes (PICO)	X	☐	6 (Lines 151-171)

BioMed Central The Open Access Publisher

Section/topic	#	Checklist item	Information reported Yes	No	Line number(s)
**METHODS**					
**Eligibility criteria**	8	Specify the study characteristics (e.g., PICO, study design, setting, time frame) and report characteristics (e.g., years considered, language, publication status) to be used as criteria for eligibility for the review	X	☐	6 (Lines 151-171)
**Information sources**	9	Describe all intended information sources (e.g., electronic databases, contact with study authors, trial registers, or other grey literature sources) with planned dates of coverage	X	☐	7 (lines 173-191)
**Search strategy**	10	Present draft of search strategy to be used for at least one electronic database, including planned limits, such that it could be repeated	X	☐	Supplementary File 1
STUDY RECORDS					
Data management	11a	Describe the mechanism(s) that will be used to manage records and data throughout the review	X	☐	8 (Line237-244)
Selection process	11b	State the process that will be used for selecting studies (e.g., two independent reviewers) through each phase of the review (i.e., screening, eligibility, and inclusion in meta-analysis)	X	☐	8 (Lines 208-222)
Data collection process	11c	Describe planned method of extracting data from reports (e.g., piloting forms, done independently, in duplicate), any processes for obtaining and confirming data from investigators	X	☐	8 (lines 238 - 310)
**Data items**	12	List and define all variables for which data will be sought (e.g., PICO items, funding sources), any pre-planned data assumptions and simplifications	X	☐	6 (Lines 150-171)
**Outcomes and prioritization**	13	List and define all outcomes for which data will be sought, including prioritization of main and additional outcomes, with rationale	X	☐	6 (Lines 150-171)
**Risk of bias in individual studies**	14	Describe anticipated methods for assessing risk of bias of individual studies, including whether this will be done at the outcome or study level, or both; state how this information will be used in data synthesis	X	☐	8 (Lines 224-235)
DATA					
**Synthesis**	15a	Describe criteria under which study data will be quantitatively synthesized	X	☐	9 (Lines 260-280)
	15b	If data are appropriate for quantitative synthesis, describe planned summary measures, methods of handling data, and methods of combining data from studies, including any planned exploration of consistency (e.g., I^2, Kendall's tau)	☐	☐	N/A
	15c	Describe any proposed additional analyses (e.g., sensitivity or subgroup analyses, meta-regression)	☐	☐	N/A
	15d	If quantitative synthesis is not appropriate, describe the type of summary planned	X	☐	10 (Lines 279-280)
**Meta-bias(es)**	16	Specify any planned assessment of meta-bias(es) (e.g., publication bias across studies, selective reporting within studies)	☐	☐	N/A

For peer review only

BioMed Central The Open Access Publisher

3

Section/topic	#	Checklist item	Information reported Yes	Information reported No	Line number(s)
**Confidence in cumulative evidence**	17	Describe how the strength of the body of evidence will be assessed (e.g., GRADE)	☐	☐	N/A

For peer review only

BioMed Central
The Open Access Publisher
